# Effects of micronutrient fortified milk and cereal food for infants and children: a systematic review

**DOI:** 10.1186/1471-2458-12-506

**Published:** 2012-07-06

**Authors:** Klaus Eichler, Simon Wieser, Isabelle Rüthemann, Urs Brügger

**Affiliations:** 1 Institute of Health Economics, Zurich University of Applied Sciences, St. Georgenstrasse, 70 P.O. Box, Winterthur 8401, CH, Switzerland

**Keywords:** Micronutrients, Fortification, Milk, Cereals

## Abstract

**Background:**

Micronutrient deficiency is a common public health problem in developing countries, especially for infants and children in the first two years of life. As this is an important time window for child development, micronutrient fortified complementary feeding after 6 months of age, for example with milk or cereals products, in combination with continued breastfeeding, is recommended. The overall effect of this approach is unclear.

**Methods:**

We performed a Systematic Review and Meta-analysis to assess the impact of micronutrient fortified milk and cereal food on the health of infants and little children (aged 6 months to 5 years) compared to non-fortified food. We reviewed randomized controlled trials using electronic databases (MEDLINE and Cochrane library searches through FEB 2011), reference list screening and hand searches. Three reviewers assessed 1153 studies for eligibility and extracted data. One reviewer assessed risk of bias using predefined forms.

**Results:**

We included 18 trials in our analysis (n = 5’468 children; range of mean hemoglobin values: 9.0 to 12.6 g/dl). Iron plus multi micronutrient fortification is more effective than single iron fortification for hematologic outcomes. Compared to non-fortified food, iron multi micronutrient fortification increases hemoglobin levels by 0.87 g/dl (95%-CI: 0.57 to 1.16; 8 studies) and reduces risk of anemia by 57% (relative risk 0.43; 95%-CI 0.26 to 0.71; absolute risk reduction 22%; number needed to treat 5 [95%-CI: 4 to 6]; 6 Studies). Compared to non-fortified food, fortification increases serum levels of vitamin A but not of zinc. Information about functional health outcomes (e.g. weight gain) and morbidity was scarce and evidence is inconclusive. Risk of bias is unclear due to underreporting, but high quality studies lead to similar results in a sensitivity analysis.

**Conclusions:**

Multi micronutrient fortified milk and cereal products can be an effective option to reduce anemia of children up to three years of age in developing countries. On the basis of our data the evidence for functional health outcomes is still inconclusive.

## Background

Micronutrient (MN) deficiency is a common public health problem, specifically for infants and children, in many low and middle income countries. For example, anemia (caused by iron deficiency) or increased infection rates and mortality (exacerbated by vitamin A and zinc deficiency) are serious threats for child development [1]. The first two years of life represent a narrow time window, which is of outstanding importance for child development [2]. During this time period future growth and vulnerable physiological capacities, such as cognitive function and motor development, are determined. Even with optimum breastfeeding, these steps depend on a an adequate quantity and quality of complementary feeding, leading to an adequate MN supply [2]. Negative health consequences resulting from suboptimal feeding, such as stunting (i.e. low height-for-age), are associated with higher morbidity and decreased function in later life [3].

Several strategies have been shown to be effective in resolving MN deficiencies for different target groups and are proposed in recommendations and guidelines [4-6]: Food based approaches (e.g. spreads to increase energy-density and MN content of food; MN powders for home fortification with sprinkles) and MN supplementation (e.g. vitamin A capsules administered at defined intervals). In addition, fortification of staple food (e.g. fortified salt, flour or oil) is widely used to resolve MN deficiencies of general populations.

Fortified complementary feeding after 6 months of age, in combination with continued breastfeeding [7], typically comprises milk or cereals products (e.g. porridge or gruel) for infants. This type of food, however, is often not covered by programs that provide fortified staple food for the general population. Primary studies have assessed the effects of fortified milk or cereals for infants and children [8,9] and some countries, such as Mexico, have introduced country wide food programs, where fortified milk is one component [10]. However, the overall evidence of the effect of fortified milk and cereals on children has not been systematically assessed.

Thus, we performed a Systematic Review to specifically assess the impact of micronutrient fortified milk and cereal food on the health of infants and children compared to non-fortified food in randomized controlled trials.

## Methods

We performed our review in accordance with current guidelines for performing [11,12] and reporting of systematic reviews [13] and established a scientific advisory board (see Acknowledgments for participating experts). A review study protocol was developed in advance, though not published.

According to our research question we defined the following inclusion criteria: **Population**: Infants and children from 6 months to 5 years of age. While our primary focus was on age groups up to 2 years, we decided to set an upper age limit at 5 years, in order not to miss suitable studies with mixed age groups. **Intervention**: Micronutrient fortified milk or cereal food. **Control intervention**: Non-fortified food; additional other nutritional approaches, if such approaches were applied in the intervention and control group. **Outcome**: At least one of the following health related outcomes: surrogate measures (such as MN serum levels, hematological parameters), functional outcome (e.g. motor development), measures of morbidity (such as disease rates) or mortality. **Study designs**: Randomized controlled trials of any follow-up time.

We excluded studies with infants and toddlers younger than 6 months [14] or applying infant formula [15], studies addressing adolescents or adult women, interventions based on supplementation, home fortification, bare food based approaches, fortification with components other than micronutrients, and studies testing absorption of MN. A priori, we also excluded studies with fortification of staple food as provided for larger population groups to isolate the effect of fortified milk and cereals.

We systematically searched for studies using electronic databases (Medline [search strategy Table 1, Cochrane library; from 1966 to February 2011; no language restriction). As this review was part of a larger project, that evaluates the economic effects of MN fortification as well, we also included search terms such as “cost” and “economics”. We screened reference lists of included papers and contacted experts in the field for additional references. In addition, we screened homepages of relevant organizations (e.g. WHO, United Nations [World Food Programme, Unicef, Millennium Development Goals], The World Bank, Pakistan National Nutrition Survey; International Clinical Epidemiology Network [16]; Global Alliance for Improved Nutrition, GAIN [17]; The Micronutrient Initiative [18]; Bill & Melinda Gates Foundation [19]). We also contacted a manufacturer (Nestlé) for further material and performed hand searches in relevant journals with developing countries issues (such as The Lancet). All references were stored in an EndNote X4 database (Thomson/ISI ResearchSoft Berkeley, CA, USA).

**Table 1:** Medline electronic search strategy

**Step**	**Search Medline 1**	**Search Medline 2**	**Search Medline 3**
1	"Infant Formula"[MeSH]^a^ OR "Milk"[MeSH]	"economics"[MeSH]	nutrition disorders[MeSH]
2	fortif*[TIAB]^b^	"micronutrients"[MeSH]	child* OR infant* OR toddl*[TIAB]
3	1 AND 2	"Nutrition Disorders"[MeSH]	"cost*"[TIAB] OR "economics"[MeSH]
4	"Cereals"[MeSH]	1 AND 2 AND3	1 AND 2 AND 3
5	fortif*[TIAB]	"cost*"[TIAB]	"india*"[TIAB] OR "pakistan*"[TIAB] OR "philippine*"[TIAB] OR "asia*"[TIAB] OR "africa*"[TIAB]
6	4 AND 5	"micronutrients"[MeSH]	4 AND 5
7	3 OR 6	"nutrition disorders"[MeSH]	
8	child*[TIAB] OR infant*[TIAB] OR toddler*[TIAB]	5 AND 6 AND 7	
9	7 AND 8	4 OR 8	

Three Medline searches were performed and retrieved references were cumulated. As this review was part of a larger project that evaluates the economic effects of micronutrient fortification as well, we included also search terms such as “cost” and “economics”.

^a^ MeSH: Medical Subject Heading.

^b^TIAB: Title/Abstract.

### Study selection and data extraction

Three reviewers screened titles and abstracts for relevance and assessed potentially relevant studies for inclusion by full text. Teaching sessions were held in advance to improve conceptual consistency between reviewers. Disagreements were resolved by consensus meetings. If data of a specific population were published in several papers or if follow-up data were presented, we included each population only once. Using a predefined form, data were extracted by one reviewer in an Excel database and checked independently by a second reviewer.

We extracted data on general study information (e.g. study region; length and completeness of follow up), study setting (e.g. level of population recruitment), population details, intervention (e.g. daily amount of fortified MN, determined as daily difference between intervention and control group; composition of MN; comparator food) and outcome (e.g. morbidity rates; hemoglobin levels [g/dl; conversion to g/L with factor 10]).

One reviewer assessed risk of bias in individual studies with a component approach exploring methodological quality on the study level (adequate generation of random sequence, concealment of allocation, blinding) as well as on the outcome level (incomplete outcome data due to attrition; selective outcome reporting) [12].

### Statistical analysis

First, we calculated pooled estimates. For continuous variables we computed weighted mean differences (WMD) and 95%-confidence intervals (CI). For example, for analysis of hemoglobin change we used the mean change in the intervention and in the control group and their pooled standard deviation (SD). If the sample size decreased during the study, we used the lower sample size at the end of the study. If mean hemoglobin change per group and SD were not reported, we calculated change as the difference of baseline and final values for intervention and control group and applied the SD of final values [20]. If 95%-CI of mean values were reported we converted them to SD assuming normal distribution [21]. To check results for robustness, we also calculated WMD for final hemoglobin values of both study groups, as this data was reported more often. Due to considerable heterogeneity between trials, we applied a random effects model [22]. When authors reported only medians for continuous data (e.g. for ferritin levels), we did not include those data in the meta-analysis. For binary data, we calculated risk ratios and 95%-CI. Heterogeneity between trials was calculated with I^2^, that is the percentage of the total variation in estimated effects that is due to heterogeneity rather than chance (where values of 25% are assigned low, 50% moderate and 75% high) [23].

Second, we divided our dataset into pre-specified subgroups to explore the influence of possible modifying factors on the outcome (fortified milk vs. cereal food; high vs. low/middle-income countries; single- vs. dual/multi-micronutrient fortification strategy).

Third, we performed a meta-regression analysis weighted for the inverse of the variance of the outcome [12]. With this approach we evaluated the unique contribution of other a priori chosen independent factors on the most often reported outcome (dependent variable: hemoglobin level; independent variables: hemoglobin levels before intervention; daily amount of fortified MN; length of follow-up; completeness of follow-up).

For parametric and non-parametric tests P-values <0.05 were considered significant. Analyses were performed using the STATA SE 9 software package (StataCorp. 2007. Stata Statistical Software, College Station, Texas, USA).

## Results

### Description of included studies

**Figure 1:**
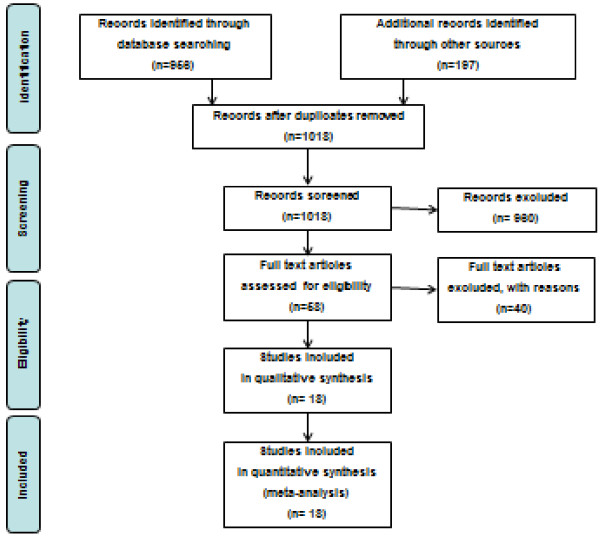
Study flow of the systematic review.

Our searches retrieved 1153 potentially relevant studies (Figure 1). Eighteen RCT [8-10,24-38] (n = 10 fortified milk; n = 8 fortified cereals) fulfilled inclusion criteria and were included for our main analysis (Table 2).

**Table 2:** Details of included studies for fortification of milk and cereal food

**Study**	**Population**	**Intervention**	**Control food**	**Outcome**	**Comment**
Author, year: Brown, 2007 [29] Design: RCT	Country: Peru Target population: periurban area; Age (mean; range): 0.6; 0.5 to 0.7 years Males (%): 46 Exclusion criteria: risk of acute malnutrition; chronic diseases	Cereals, fortified (porridge); single MN strategy MN applied ^a^: Zn Iron dosage ^b^: n.a.^c^ mg/day; Iron compound ^d^: n.a.	porridge, unfortified for zink	After 0.5 year: plasma zinc; anthropometry; infections	Both groups recieved iron fortification and vitamin supplements, thus net intervention was zinc fortification.
Author, year: Daly, 1996 [31] Design: RCT	Country: UK (74% white; 24% Afro-Caribbean; 2% Asian) Target population: poor innerurban Age (mean; range): 0.65; 0.5 to 0.7 years Males (%): 47 Exclusion criteria: preterm at birth	Milk, fortified; multi MN strategy MN applied: Fe, VitA, other Vitamins, other MN Iron dosage: 5.47 mg/day; Iron compound: no info	milk, unfortified	After 1 year: hematological parameters; anthropometry	Functional outcome was extracted from related paper Williams_1999 [39].
Author, year: Faber, 2005 [33] Design: RCT	Country: South Africa Target population: rural area, low socio-economic status, Age (mean; range): 0.7; 0.6 to 0.9 years Males (%): 51 Exclusion criteria: birth weight <2500 g, severe anemia	Cereals, fortified (porridge); multi MN strategy MN applied: Fe, Zn, other Vitamins Iron dosage: 27.5 mg/day; Iron compound: FeFu	porridge, unfortified	After 0.5 year: hematological parameters, serum retinol, zinc; growth; motor development	Population baseline characteristics only for infants who completed the study.
Author, year: Gibson, 2011 [35] Design: RCT	Country: Zambia Target population: middle income class Age (mean; range): 0.5; 0.5 to 0.5 years Males (%): 48 Exclusion criteria: "not in good health"	Cereals, fortified (porridge); multi MN strategy MN applied: Fe, Zn, other Vitamins, other MN Iron dosage: 5.36 mg/day; Iron compound: no info	porridge, unfortified	After 1 year: hematological parameters; serum zink, anthropometry; hospital referral; death; diarrhea; pneumonia; mental and motor development	All children received VitA and Iodine by a public supplementation program. Some outcomes extracted from related paper (Chilenje_2010) [40] and (Manno_2011) [41].
Author, year: Gill, 1997 [24] Design: RCT	Country: Ireland Target population: no info Age (mean; range): 0.5; 0.5 to 0.5 years Males (%): 51 Exclusion criteria: severe or chronic disaese, malnutrition; congenital anomalies	Milk, fortified; single MN strategy MN applied: Fe Iron dosage: 6.54 mg/day; Iron compound: FeSu	formula milk, unfortified for iron	After 0.75 year: hematological parameters, anthropometry	
Author, year: Lartey, 1999 [34] Design: RCT	Country: Ghana Target population: urban area Age (mean; range): 0.5; 0.5 to 0.5 years Males (%): 48 Exclusion criteria: congenital abnormalities	Cereals, fortified (porridge); multi MN strategy MN applied: Fe, Zn, VitA, other Vitamins, other MN Iron dosage: 14.25 mg/day; Iron compound: electrFe	porridge, unfortified	After 0.5 year: hematological parameters; anthropometry; diarrhea; fever; respiratory illness	Intervention cereal with 2 formulations of fortification depending on daily cereal intake of infant to avoid potential toxicity problems.
Author, year: Liu, 1993 [37] Design: RCT	Country: China Target population: all population classes (90% of all children) Age (mean; range): 0.8; 0.5 to 1.1 years Males (%): 55 Exclusion criteria: no info	Cereals, fortified (rusk); multi MN strategy MN applied: Fe, Zn, VitA, other Vitamins, other MN Iron dosage: 5 mg/day; Iron compound: FeAmCi	rusk, unfortified	After 0.25 year: hematological paramters; MN-serum levels, anthropometry	
Author, year: Maldonado Lonzano, 2007 [25] Design: RCT	Country: Spain Target population: no info Age (mean; range): 1.9; (range: no info) years Males (%): 58 Exclusion criteria: iron supplementation	Milk, fortified; multi MN strategy MN applied: Fe, other Vitamins, other MN Iron dosage: 5.9 mg/day; Iron compound: no info	milk, unfortified (cows whole milk formula)	After 0.33 year: hematological parameters	No child with anemia at baseline.
Author, year: Morley, 1999 [26] Design: RCT	Country: UK (Indian ethnicity) Target population: mother with higher eduction, non-manual social class Age (mean; range): 0.78; (range: no info) years Males (%): 50 Exclusion criteria: relevant disease; iron supplementation	Milk, fortified; single MN strategy MN applied: Fe Iron dosage: 1.8 mg/day; Iron compound: FeSu	formula, unfortified	After 0.75 year: hematological parameters, antropometry, motor and mental development	Only data from Norwich cohort blood samples could be taken at baseline and were extracted for Hb outcome.
Author, year: Nesamvuni, 2005 [36] Design: RCT	Country: South Africa Target population: poor socio-economic status, undernourished children Age (mean; range): no info; 1 to 3 years Males (%): 0 Exclusion criteria: physical or mental disability, severe undernutrition	Cereals, fortified (maize porridge); dual MN strategy MN applied: VitA, other Vitamins Iron dosage: n.a.mg/day; Iron compound: n.a.	maize meal, unfortified	After 1 year: hematological parameters, retinol level, anthropometry	Children and family members received the food.
Author, year: Oelofse, 2003 [9] Design: RCT	Country: South Africa Target population: urban disadvantaged black community (low socioeconomic status) Age (mean; range): 0.5; (range: no info) years Males (%): 0 Exclusion criteria: birth weight < 2.5 kg; congenital abnormalities	Cereals, fortified (porridge); dual MN strategy MN applied: Zn, other Vitamins Iron dosage: -0.8 mg/day; Iron compound: FePP	normal diet	After 0.5 year: hematological parameters, zinc level, retinol level, anthropometry, psychomotor development	90% of control group already recieved commercially prepared complementatry food. The food concentration of iron did not relevanlty differ between groups, but of Zinc and of VitA.
Author, year: Rivera, 2010 [10] Design: RCT (accounted for cluster randomisation)	Country: Mexico Target population: households living in poverty Age (mean; range): no info; 1 to 2.5 years Males (%): 50 Exclusion criteria: no info	Milk, fortified; multi MN strategy MN applied: Fe, Zn, other Vitamins, other MN Iron dosage: 7.82 mg/day; Iron compound: FeGlu	milk, non-fortified	After 1 year: hematological parameters	Study results are adjusted for cluster effect. Evaluation of a large scale program (Leche Lincosa) in Mexico.
Author, year: Sazawal, 2010 [8] Design: RCT	Country: India Target population: periurban area; illiteracy of parents Age (mean; range): 1.9; 1 to 3 years Males (%): 50 Exclusion criteria: severe malnutrition; severe illness	Milk, fortified; multi MN strategy MN applied: Fe, Zn, VitA, other Vitamins, other MN Iron dosage: 8.3 mg/day; Iron compound: FeSu	milk, unfortified	After 1 year: hematological parameters, anthropometry, severe illnesses, diarrhoea, lower respiratory tract infections, pneumonia	Some data extracted from relating paper: Sazawal_2006 [42] Completeness relates to hematologic parameters.
Author, year: Schümann, 2005 [38] Design: RCT	Country: Guatemala Target population: low income; periurban settlement Age (mean; range): 1.7; 1 to 2 years Males (%): 52 Exclusion criteria: gastric or intestinal diseases; infections	Cereals, fortified (bean paste); single MN strategy MN applied: Fe Iron dosage: 17.1 mg/day; Iron compound: FeSu	beans, unfortified	After 0.19 year: hematological parameters	All children recieved anthelmintic treatment; all families were compensated. Three arm trial: Only data for FeSu group (n = 31) vs. control group (n = 30) extracted.
Author, year: Stevens, 1998 [32] Design: RCT	Country: UK (mostly caucasian) Target population: lower social classes were overrepresented Age (mean; range): 0.5; 0 to 0 years Males (%): 0 Exclusion criteria: illness, major congenital malformation	Milk, fortified; single MN strategy MN applied: Fe Iron dosage: 6.87 mg/day; Iron compound: FeSu	milk, unfortified	After 1 year: hematological parameters	
Author, year: Villalpando, 2006 [27] Design: RCT	Country: Mexico Target population: poor periurban community Age (mean; range): 1.8; 0.8 to 2.5 years Males (%): 50 Exclusion criteria: no info	Milk, fortified; multi MN strategy MN applied: Fe, Zn, other Vitamins Iron dosage: 6.74 mg/day; Iron compound: FeGlu	milk, unfortified	After 0.5 year: hematological parameters	The results of the study lead to broadening of a fortified milk distribution program in Mexico.
Author, year: Virtanen, 2001 [28] Design: RCT	Country: Sweden Target population: urban area Age (mean; range): 1; 1 to 1 years Males (%): 39 Exclusion criteria: milk intolerance; poor health	Milk, fortified; single MN strategy MN applied: Fe Iron dosage: 4.53 mg/day; Iron compound: FeGlu, FeLac	milk, unfortified	After 0.5 year: hematological parameters	
Author, year: Walter, 1998 [30] Design: RCT	Country: Chile Target population: From four contiguos urban communities Age (mean; range): 0.5; 0 to 0 years Males (%): 52 Exclusion criteria: major birth or neonatal complications, chronic illness	Milk, fortified; single MN strategy MN applied: Fe Iron dosage: 6.5 mg/day; Iron compound: FeSu	formula, low iron fortifed	After 1 year: hematological parameters, anthropometry	

^a^ MN (micronutrient) applied: Fe: iron; Zn: zinc; VitA: Vitamin A; other Vitamins: e.g. Vitamin C; other MN (micronutirents): e.g. selen, copper.

^b^ Iron dosage: Determined as daily difference between intervention and control group.

^c^ n.a.: not applicable

^d^ Iron compound: FeSu: iron-sulfate; FePP: iron-pyrophosphate; NaFeEDTA: natrium-iron-EDTA; FeFu: iron-fumarate; FeGlu: iron-gluconate; FeAmCi: ferric-ammonium-citrate; FeLa: Ferrous lactate; electrFe: electrolytic iron.

These 18 trials comprised 5468 infants and children from different regions (2 studies from Asia [8,37], 5 studies from Africa [9,33-36]; 5 Studies from South- and Middle-America [10,27,29,30,38]; 6 Studies from Europe [24-26,28,31,32]).

Study population sizes varied from n = 33 to n = 1120 participants (median 166; IQR 92 to 361). Most participants belonged to vulnerable groups and had been recruited from different settings (8 studies: medical or community care centers:, 7 studies: low income risk groups; 2 studies: general population of peri-urban and rural areas; 1 study: no information given). The most frequent exclusion criteria were chronic diseases, severe anemia, severe mal-/under-nutrition, and low birth weight. Mean age of participants ranged from 6 to 23 months at inclusion (upper age limit was 3 years in one study [8]) and the sex ratio was well balanced. Mean hemoglobin values of children at baseline varied between studies from 9.0 g/dl to 12.6 g/dl (median of study values: 11.1 g/dl). Follow up periods were generally short and did not exceed one year (mean follow up: 8.2 months; range: 2.3 to 12).

Fortified milk was prepared with centrally processed fortified milk powder in most of the studies. Fortified cereals comprised centrally processed weaning or complementary food, such as fortified porridge, gruel or weaning rusk to prepare a pap. Iron was the most frequently used MN for fortification (15 of 18 trials), followed by zinc (9 trials) and vitamin A (6 trials). Seven studies used a single-MN fortification strategy (6 studies with iron only; 1 study with zinc only), two studies a dual- and 9 studies a multi-micronutrient (MMN) strategy (i.e. 3 or more MN, for example additional fortification with vitamin C and E, selenium, copper).

### Effect on hemoglobin levels

**Figure 2:**
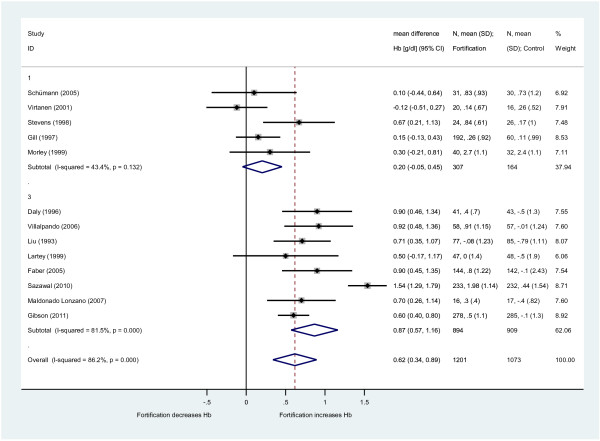
**Effect of iron fortification of milk and cereals on hemoglobin (Hb) levels compared to non-fortified food.** Only studies with iron fortification included (n = 13 RCT). Results are provided as weighted mean difference in hemoglobin (WMD: g/dl with 95%-CI; conversion to g/L with factor 10) between intervention and control group (iron single-fortification (1); iron multi micronutrient fortification (3); overall effect).

Hemoglobin blood level was the most frequently reported outcome parameter. Across 13 studies that tested iron fortification irrespective of other added MN, the mean increase of hemoglobin compared to the control group was 0.62 g/dl (95%-CI: 0.34 to 0.89) for children fed with fortified milk or cereals (Figure 2). Heterogeneity was high (I^2^ = 86%). Comparison of different subgroups showed a stronger effect of the iron MMN fortification approach (n = 8 studies; hemoglobin increase 0.87 g/dl (95%-CI: 0.57 to 1.16; I^2^ = 82%) compared to the iron single-fortification strategy (n = 5 studies; hemoglobin increase 0.20 g/dl (95%-CI: -0.05 to 0.45; I^2^ = 43%). The daily applied iron dosage was similar for the single-iron approach (median: 6.5 mg) and the MMN-approach (median 6.7 mg).

### Effect on anemia prevalence

**Figure 3:**
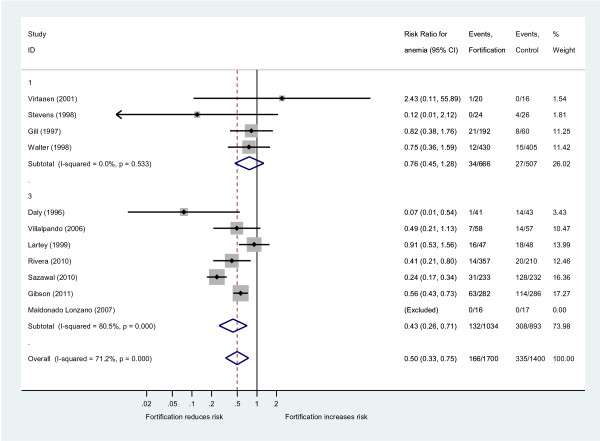
**Effect of iron fortification of milk and cereals on anemia compared to non-fortified food.** Only studies with iron fortification included (n = 11 RCT). Results are provided as risk ratio (RR, 95%-CI) of suffering from anemia in the intervention group compared to the control group (iron single-fortification (1); iron multi micronutrient fortification (3); overall effect).

Eleven trials provided data for anemia rates, all of them using iron as a single- or a MMN-fortification strategy. Applied thresholds for anemia varied between 10.5 g/dl and 11 g/dl and the median of anemia rates at baseline was 36% (IQR: 15% to 40%; 9 studies with data). Fortified milk or cereals reduced the risk of suffering from anemia by 50% (risk ratio 0.50, 95%-CI: 0.33 to 0.75; I^2^ = 71%; Figure 3). Again, a stronger effect of the MMN fortification approach emerged (n = 7 studies; risk ratio 0.43 (95%-CI: 0.26 to 0.71; I^2^ = 81%) compared to the iron single-fortification strategy (n = 4 studies; risk ratio 0.76 (95%-CI: 0.45 to 1.28; I^2^ = 0%). Overall, the absolute risk reduction (ARR) of suffering from anemia was 14% (un-weighted data of 11 trials), translating into a number needed to treat (NNT) with fortified milk or cereals of 7 (95%-CI: 6 to 9) participants over a period of 8 months (i.e. the mean follow-up time) to avoid one case of anemia. For the MMN approach these results are even more favorable (un-weighted data of 7 trials: ARR 22%; NNT 5 [95%-CI: 4 to 6]).

### Effect on ferritin levels

Ferritin is the most direct measure to conclude if iron stores increase by iron-fortified food consumption. Eleven trials provided data for ferritin serum levels. Ferritin levels were not adjusted for subclinical infections. Given the skewed distribution of ferritin values, authors often reported median estimates. Medians were significantly higher in the intervention groups (ranges of ferritin medians at end of study [micro-g/l]: intervention: 15.8 to 44.6; control: 6.5 to 28; P < 0.01). Only three studies provided mean values to be included in a meta-analysis, which showed an effect in the same direction. The mean ferritin increase with iron fortification was 11.3 micro-g/l (95%-CI: 3.3 to 19.2; I^2^ = 79%) compared to control groups.

### Effects on serum zinc and vitamin A levels

Five studies provided data for change in serum zinc levels. MN fortification with zinc led to no relevant change in zinc serum levels (0.4 micro-g/dl (95%-CI: -1.7 to 2.6; I^2^ = 0%) compared to control groups. However, fortification increased vitamin A serum levels compared to control groups (four studies with data: Retinol increase by 3.7 micro-g/dl [95%-CI: 1.3 to 6.1; I^2^ = 37%]).

### Effects on growth, functional measures and morbidity

For three European studies, no relevant effect of fortification on height and weight was seen and morbidities were not an issue in this population.

All other results relate to non-European low-/middle income countries. Due to the short follow-up period in most of the studies, no meaningful conclusion can be drawn for possible effects of fortification on height or weight gain or z-scores (weight-for-age; height-for-age; weight-for-height). Of 9 studies with data, 7 trials reported no relevant differences between intervention and control group at the end of the study. In one study [36], more weight gain was seen in the intervention group after one year (4.6 kg vs. 2.0 kg; P < 0.05), in another study [8] children consuming fortified milk showed improvement in weight gain compared to control group (difference 0.21 kg/year [95%-CI 0.12 to 0.31) and height gain (difference 0.51 cm/year [95%-CI 0.27 to 0.75).

Of three studies with data for psychomotor development of children, two trials reported no relevant difference between groups [9,35] and one study [33] found slight improvements compared to the control group.

Of four studies with morbidity data of children, three trials reported no relevant differences between groups for infections [29], for diarrhea, fever and respiratory illness [34] and for referral to hospital or death in partly HIV exposed children [35]. In one study [8] fortified milk significantly reduced the probability of days with severe illness (by 15%), and the relative risk of diarrhea (by 18%) and lower respiratory illness (by 26%).

### Exploring heterogeneity

In our pre-specified subgroup analyses no relevant influence on the outcome was detected for the mode of fortified food (fortified milk vs. cereals). Hemoglobin change was somewhat higher in studies from low/middle income countries (0.78 g/dl (95%-CI: 0.41 to 1.15) compared to high income countries (0.42 g/dl (95%-CI: 0.10 to 0.73), but the difference was not statistically significant. The dual-/multi-micronutrient approach led to a significantly stronger effect of iron fortification on hemoglobin increase than the iron single-fortification strategy (Figure 3).

In our multivariable meta-regression analysis, none of the tested independent variables (mean hemoglobin level before intervention; daily amount of consumed iron, length of follow-up, completeness of follow up) was significantly associated with the change in hemoglobin.

### Summary assessment of risk of bias

Only two [8,25] of 18 trials provided enough information to conclude that both random sequence generation and allocation concealment was adequately performed (Table 3). For 11 trials this was unclear and inadequate procedures had been applied in 5 trials. Other criteria were fulfilled more often: Blinding was reported in 13 of 18 studies, incomplete outcome data were addressed in 8 of 18 trials and 12 of 18 studies showed no selective outcome reporting (i.e. besides serum markers also height/weight, functional measures or morbidities were reported).

**Table 3:** Risk of bias summary table

**EN**	**Author**	**Year**	**Adequate sequence generation?**	**Allocation concealment?**	**Blinding?**	**Incomplete outcome data addressed**	**Are typical outcomers reported? (Selective outcome reporting)**
675	Brown	2007	?	?	YES	YES	YES
818	Daly	1996	?	?	?	YES	YES
951	Faber	2005	NO	NO`	YES	NO	YES
1058	Gibson	2011	?	?	YES	NO	YES
153	Gill	1997	?	YES	NO	NO	YES
1051	Lartey	1999	?	YES	NO	YES	YES
1154	Liu	1993	?	?	?	NO	YES
257	Maldonado Lonzano	2007	YES	YES	YES	YES	NO
282	Morley	1999	?	YES	YES	YES	YES
1149	Nesamvuni	2005	NO	NO	YES	YES	YES
297	Oelofse	2003	NO	NO	NO	NO	YES
333	Rivera	2010	?	?	YES	NO	NO
1	Sazawal	2010	YES	YES	YES	NO	YES
1172.2	Schumann	2005	NO	NO	YES	YES	NO
838	Stevens	1998	?	?	YES	NO	NO
403	Villalpando	2006	?	?	YES	YES	NO
404	Virtanen	2001	?	?	YES	NO	NO
797	Walter	1998	NO	YES	YES	NO	YES

The table presents each study by assessed methodological criterion in a cross-tabulation ^a^. Studies are sorted for author name.

^a^ Assessment categories: YES: criterion fulfilled; NO: criterion not fulfilled; “?”: unclear, as no information given.

In summary, the risk of bias for the most often reported outcomes hemoglobin change and anemia rates is unclear. However, a sensitivity analysis including only studies with low risk of bias led to similar results (three studies that fulfilled 4 of 5 quality criteria [8,25,26]: hemoglobin increase 0.87 g/dl (95%-CI: 0.09 to 1.65; I^2^ = 92%). Another sensitivity analysis showed that the result pattern remained basically unchanged after performing analyses using mean values of groups at the end of the study, instead of mean changes of groups.

## Discussion

To our knowledge, this is the first systematic review, that has applied a meta-analysis to specifically weigh the overall evidence for the effects of fortified milk and cereal food suitable for complementary feeding of children. The evidence relates to study populations between 6 month and three years of age. Iron fortification leads to a clinically relevant increase in hemoglobin levels and reduction of anemia rates. For zinc and vitamin A fortification only surrogate parameters are reported, but the combination with iron (MMN approach) leads to a more pronounced effect on hemoglobin levels compared to an iron single-fortification strategy. The evidence for functional health outcomes is inconclusive.

### Strengths and limitations

We applied a thorough search strategy with a stepwise retrieval of studies using electronic databases and additional sources. We cannot exclude having missed references but we believe that we found a near complete sample of relevant papers for our specific research question.

Some limitations have to be mentioned. First, included studies showed short follow-up periods, thus the impact of fortified milk or cereal food on functional health outcomes (such as sustainable height and weight gain or mental and motor development) could not be assessed thoroughly. Second, for zinc and vitamin A fortification only surrogate outcomes as serum levels are available. However, the presence of additional fortified micronutrients seems to be important for the effects of iron on hemoglobin levels. The MMN approach is more effective than iron single fortification in our review, reflecting that complex micronutrient deficiencies are responsible for health problems [20]. Third, risk of bias is unclear mainly due to underreporting of the randomization procedure and incomplete outcome data. Finally, pooled estimates have to be interpreted cautiously as statistical heterogeneity between studies was considerable and meta-regression did not reveal significant associations of pre-specified study characteristics with study results. Possible sources for unexplained heterogeneity might be underreporting for co-interventions (e.g. public supplementation or food programs) or the diversity of applied MN preparations that have influence on MN absorption. For example, five different iron compounds were used (12 studies with data: six times FeSulfate; twice FeGluconate; one time, each, FePyrophosphate; FeFumarate; Ferric ammonium citrate; electrolytic iron). In addition, the difference in daily consumed iron between intervention and control group varied between 1.8 mg and 14.3 mg. Furthermore, molar ratios, a determinant for MN absorption, also showed variation (ranges of molar ratios: ascorbic acid/iron: 0.68 to 30; phytic acid/iron: 1.7 to 2.2; calcium/iron: 40 to 134).

### Existing systematic reviews and research needs

Important contributions have been made in the recent years with other systematic reviews to evaluate the health effects of MN interventions. These reviews differ from ours: Dewey and Adu-Afarwuah gave a broad systematic overview of studies and programs aimed at improving biochemical and functional outcomes with complementary foods [43]. However, they did not perform a meta-analysis and presented results in a tabulated form or as averaged effect sizes. Some reviews have concentrated on MN supplementation only [44-48] or home fortification [49], other reviews have combined supplementation and fortification strategies for analysis [20,50], included children as well as adolescents or adult women [6,51,52], or included fortified staple food interventions [6,52].

The health effects found in our review are in line with effect sizes shown in some similar reviews above [20,47,52]. This underpins the validity of our findings and supports a strategy to intervene with fortified milk and cereal food for infants and children. Supplementation trials, for example with vitamin A, have been shown to reduce mortality and morbidity via improved nutritional status [47,48], even though serum level increases were small [53], similar to our review. Thus, some authors conclude that fortification would also have an impact on morbidity and mortality, although a conclusive answer cannot yet be given [52]. On the other hand, negative aspects of iron supplementation have been reported, such as increased morbidity and mortality in regions where malaria transmission is intense [54]. Thus, recommendations concerning iron supplementation have been formulated [55]. These adverse effects, however, may not be that relevant for fortified foods. Daily micronutrient dosages of fortified foods are much lower as compared to supplementation. Furthermore, children stop eating once they get saturated, which may also not be the case for high dosage sprinkles, that can be seen as a specific application of supplementation. Nevertheless, long term data concerning negative effects of iron fortified foods in regions with high prevalence of malaria and infectious diseases are lacking.

Further compiled evidence is needed to agree on the optimal MN preparation for fortified milk and cereals (such as composition of MN; suitable compounds; molar ratios for additives) to fully exhaust the potential of this approach. Future studies should also focus on health outcomes of MN fortification beyond the effect of iron on hematological results, for example via long-term follow-up of study populations.

### Implications for decision makers

There are multiple delivery mechanisms for fortified milk and cereal food. Production and distribution via government programs and local public agencies would be an obvious option to strengthen local structures. Implementation of effective strategies, however, does not always work well in the field due to logistical problems or inappropriate priority setting [56]. Thus, some have discussed the role of the business community in improving nutrition in developing countries [56-58]. Commercially distributed fortified foods (e.g. with iron) are already available in many markets, even in low-income countries. In a public private partnership, business partners can provide their professional knowledge and experience concerning technical problems with processing and fortification, supply and transport, or refrigeration and conservation issues (specifically important for milk) to get interventions more efficient.

A limitation of the market approach is that it may not reach the poorest of the poor. Thus, a combination of different delivery channels, as well as affordable prices, may be needed. Children with severe anemia, who may be overrepresented in very poor groups, are often excluded from trials due to ethical reasons. One may assume, that the positive effects on the hemoglobin levels may be even stronger for such children. Additional economic analyses are necessary to contribute to a deeper understanding of the health economic effects of such a strategy and to inform the priority setting of decision makers.

## Conclusions

Multi micronutrient fortified milk and cereal products can be an effective option to reduce anemia of children up to three years of age in developing countries. On the basis of our data the evidence for functional health outcomes is still inconclusive.

## Abbreviations

Fe, Iron; MN, Micro nutrients; MMN, Multi micro nutrients; RCT, Randomized controlled trial; WMD, Weighted mean difference.

## Competing interests

The authors declare that they have no competing interests.

## Authors’ contributions

KE designed and conducted research, analyzed data and drafted the manuscript. SW helped to design research, conducted research, helped to draft the manuscript. IR conducted research, helped to draft the manuscript. UB helped to design research, helped to draft the manuscript. All authors read and approved the final manuscript.

## Expert and Affiliation

Lindsay H. Allen - Western Human Nutrition Research Center, USA; Narendra K. Arora - International Clinical Epidemiology Network, INCLEN, India; Zulfiqar A. Bhutta - Aga Khan University, Pakistan; Rodolfo F. Florentino - Nutrition Foundation of the Philippines; Guillermo Meléndez - Mexican Health Foundation, Mexico; Noel W. Solomons - Program Director for Central America, International Nutrition Foundation, Guatemala; Edgar Vasquez-Garibay - University of Guadalajara, Mexico.

## Pre-publication history

The pre-publication history for this paper can be accessed here:

http://www.biomedcentral.com/1471-2458/12/506/prepub
